# A smart watch crosses the threshold into clinical practice—or not quite yet?

**DOI:** 10.1007/s44470-026-00195-4

**Published:** 2026-10-05

**Authors:** Rami N. Khayat, Matthew S. Floyd

**Affiliations:** 1 Division of Pulmonary, Allergy, Critical Care and Sleep Medicine, Pennsylvania State University, 500 University Drive, Mail Code H041, Rm C5800, PO Box 850, Hershey, PA 17033 USA; 2 The Penn State Health Sleep Center, Pennsylvania State University, 1214 West End Ave, Suite 1159, Hummelstown, PA 17036 USA

Sleep, a quantifiable and clinically rich physiological state, is a natural target for continuous monitoring and investigation by health technology. Since the introduction of wrist-worn actigraphy by Kripke and colleagues [1], interest in wearable sleep monitors has grown steadily. Despite the rapid evolution of wearable technology, the development of standards to validate these technologies has not kept pace. As consumer wearables increasingly engage in delivering diagnostic assessment and medical advice, experts are calling for standards and guidelines for the testing and applications of these devices [2].

Notwithstanding the gap in testing and validation, consumer adoption has reached a critical tipping point over the past few years with over 40% of US adults now utilizing wearable devices [3]. As sensor capabilities and multimodal analytics continue to mature, the clinical utility of wearable technology in health monitoring and disease management is poised for substantial growth. Beyond detection of the sleep–wake state, the addition and enhancement of photoplethysmography, oximetry sensors, and algorithms have enabled consumer wearable to assess cardiorespiratory parameters and suggest the presence of obstructive sleep apnea (OSA).

In their study recently published in the Journal of Clinical Sleep Medicine, Alavi et al. [4] detail a rigorous validation and clinical testing study they performed on a wearable device (Samsung Galaxy Watch) that was recently granted FDA clearance to detect moderate to severe OSA over a two-night monitoring period. This feature uses photoplethysmography, heart rate, and proprietary “sleep pattern information” to detect OSA. While the authorization did not allow the company “to assist clinicians in diagnosing sleep disorders,” the algorithm does generate an estimated AHI (“eAHI”). Regardless of the motive for seeking initial FDA approval, it is an encouraging development to see that industry is seeking FDA approval for their devices’ clinical claims. Alavi et al. set out to test the claim, while also attempting to provide clinical context for use of the device.

The study evaluated 147 participants on two separate nights, gathering data from both in-lab polysomnography and the Galaxy Watch; the sampled nights were separated by four to ten at-home nights. This design allowed direct alignment with the FDA-cleared claim of a two-night decision framework for the tested watch while capturing night-to-night physiological variability. The investigators compared the eAHI against PSG-derived respiratory indices and PSG-derived hypoxic burden-defined risk strata. The watch-derived eAHI exhibited its strongest diagnostic performance when evaluated against OAHI 4% (AUROC = 0.94, *r* = 0.87). This aligns with a more “hypoxia-based” screening approach of the wearable technology and highlights a potentially low discriminatory value for patients with more mild disease or a more “arousal-based” impact as is expected with the OAHI 3%.

A conceptual strength in this study is benchmarking device performance, not only against conventional event-frequency metrics, but also against the emerging variable of hypoxia burden (HB) derived from the PSG. For participants categorized into the high-risk HB stratum, the watch algorithm achieved 100% sensitivity and 100% specificity. This demonstrates that optical photoplethysmography (PPG) and wrist oximetry are especially discriminative when the cumulative physiological hypoxemic stimulus is large. The authors conducted structured subgroup risk ratio analyses across sex, BMI, age, ethnicity, and objective skin pigmentation metrics. They demonstrated consistent sensitivity (> 91%) and specificity across darker skin tones addressing a known vulnerability of optical pulse oximeters.

Despite rigorous testing analysis, several concerns remain. Most importantly, the study population was enriched for individuals with either a prior diagnosis of moderate-to-severe OSA or a high pre-test probability (STOP-Bang ≥ 3). Of the 147 completing subjects, 97 participants had moderate-to-severe OSA on both nights, and only 29 had an AHI < 15 on both nights. This spectrum bias artificially inflates positive predictive values (PPVs exceeding 95%). At the manufacturer’s default eAHI threshold of 15 events/h, the device exhibited a high sensitivity (94.1%) but a notably modest specificity of only 66.7% against OAHI 4% (and 65.8% against AHI 4%). The low specificity stands in contrast to the 87.7% specificity in comparison to the FDA De Novo summary performance. To achieve high specificity (94.9%), the cutoff was shifted to a cohort-optimized threshold of 25.95 events/h (which reduced sensitivity to 82.4%). Our ability to superimpose these performance metrics to a low-prevalence, general screening population is limited as a result. In a low-prevalence population, one would expect the false positive rate to be substantially higher.

Investigators did note limitations in valid data collection. Among the 147 participants who completed both in-lab PSG sessions, 57 participants lacked valid watch eAHI data on one or both in-lab nights due to poor signal quality, motion artifact, or inadequate wrist coupling. The authors supplemented missing data with home recordings to offset this limitation for research purposes. Unwitnessed home recordings remain susceptible to unmeasured confounders (e.g., positional therapy, alcohol consumption, sleep fragmentation, or undiagnosed central events). A wearable data invalidation rate of 38.8% during an attended polysomnogram highlights the ongoing real-world challenge of sensor displacement and optical signal loss in wrist-worn consumer hardware.

Finally, the study excluded patients with significant cardiac disease, chronic pulmonary disease, movement disorders, and comorbid sleep disorders. Because these comorbid conditions are common in patients referred for sleep evaluation, the performance of the watch algorithm in multimorbid populations remains unproven.

In clinical practice, the use of this device as a sole screening tool, operating at the default 15 events/h threshold, would generate a significant number of false positives, which may burden downstream sleep clinic resources and confirmatory diagnostic pathways. Clinicians must understand that at default thresholds, lower specificity (66.7%) will still necessitate clear clinical workflows and confirmatory diagnostic testing (PSG, HSAT) before initiating therapy. The US Preventive Services Task Force does not currently support the screening of OSA in the absence of symptoms, due to insufficient evidence of screening accuracy and inconsistent evidence on health outcomes [5]. However, the evaluated device can perform well at identifying high-risk individuals with significant hypoxic burden, serving as an accessible triage entry point.

In our view, their work by Alavi et al. provides a reasonable roadmap for addressing the validation and clinical context of emerging wearable devices and algorithms. Deployment of this device would still require careful appraisal of positive and negative predictive values within the specific population and setting.

## Data Availability

Not applicable.

## References

[1] Kripke DF, Mullaney DJ, Messin S, Wyborney VG. Wrist actigraphic measures of sleep and rhythms. Electroencephalogr Clin Neurophysiol. 1978;44(5):674–6. 10.1016/0013-4694(78)90133-5. 77772

[2] Chiang AA, Khosla S. Consumer wearable sleep trackers: are they ready for clinical use. Sleep Med Clin. 2023;18(3):311–30. 10.1016/j.jsmc.2023.05.005. 37532372

[3] Nagappan A, Krasniansky A, Knowles M. Patterns of ownership and usage of wearable devices in the United States, 2020-2022: survey study. J Med Internet Res. 2024;26:e56504. 10.2196/56504. PMC11316147 39058548

[4] Alavi A, Costa E, Matsumoto MM, Odenwald N, Elkarra N, Ma Y, et al. Smartwatch-based detection of moderate-to-severe and high-risk obstructive sleep apnea. J Clin Sleep Med. 2026;22(1):150. 10.1007/s44470-026-00159-8. PMC13522277 42661136

[5] Mangione CM, Barry MJ, Nicholson WK, Cabana M, Chelmow D, Rucker Coker T, et al.; US Preventive Services Task Force. Screening for obstructive sleep apnea in adults: US Preventive Services Task Force recommendation statement. JAMA. 2022;328(19):1945–50. 10.1001/jama.2022.20304. 36378202

